# Regulation of Sialidase Production in *Clostridium perfringens* by the Orphan Sensor Histidine Kinase ReeS

**DOI:** 10.1371/journal.pone.0073525

**Published:** 2013-09-04

**Authors:** Thomas J. Hiscox, Paul F. Harrison, Anjana Chakravorty, Jocelyn M. Choo, Kaori Ohtani, Tohru Shimizu, Jackie K. Cheung, Julian I. Rood

**Affiliations:** 1 Department of Microbiology, Monash University, Clayton, Victoria, Australia; 2 Victorian Bioinformatics Consortium, Monash University, Clayton, Victoria, Australia; 3 Department of Microbiology, Graduate School of Medical Science, Kanazawa University, Takara-machi Kanazawa, Ishikawa, Japan; Oregon State University, United States of America

## Abstract

*Clostridium perfringens* is ubiquitous in nature and is often found as a commensal of the human and animal gastrointestinal tract. It is the primary etiological agent of clostridial myonecrosis, or gas gangrene, a serious infection that results in extensive tissue necrosis due to the action of one or more potent extracellular toxins. α-toxin and perfringolysin O are the major extracellular toxins involved in the pathogenesis of gas gangrene, but histotoxic strains of *C. perfringens*, such as strain 13, also produce many degradative enzymes such as collagenases, hyaluronidases, sialidases and the cysteine protease, α-clostripain. The production of many of these toxins is regulated either directly or indirectly by the global VirSR two-component signal transduction system. By isolating a chromosomal mutant and carrying out microarray analysis we have identified an orphan sensor histidine kinase, which we have named ReeS (regulator of extracellular enzymes sensor). Expression of the sialidase genes *nanI* and *nanJ* was down-regulated in a *reeS* mutant. Since complementation with the wild-type *reeS* gene restored *nanI* and *nanJ* expression to wild-type levels, as shown by quantitative reverse transcription-PCR and sialidase assays we concluded that ReeS positively regulates the expression of these sialidase genes. However, mutation of the *reeS* gene had no significant effect on virulence in the mouse myonecrosis model. Sialidase production in *C. perfringens* has been previously shown to be regulated by both the VirSR system and RevR. In this report, we have analyzed a previously unknown sensor histidine kinase, ReeS, and have shown that it also is involved in controlling the expression of sialidase genes, adding further complexity to the regulatory network that controls sialidase production in *C. perfringens*.

## Introduction

The Gram-positive spore-forming anaerobe *Clostridium perfringens* is ubiquitous in soil and sewage and is a commensal of the human and animal gastrointestinal tract [1], [2]. In addition, *C. perfringens* is the causative agent of human and animal diseases such as clostridial myonecrosis (gas gangrene), food poisoning and several enterotoxaemia and enteritis syndromes [3], [4]. The key feature of these diseases is that they are mediated by one or more of the potent toxins produced by *C. perfringens* [1], [5].

Clostridial myonecrosis results from the contamination, by *C. perfringens* type A cells or spores, of wounds that result from either traumatic injury or gastrointestinal surgery [3]. The major toxins involved in the disease are α-toxin and perfringolysin O [6], [7]. α-toxin is essential for virulence and perfringolysin O, although not essential, has a synergistic role in the disease process [6], [7]. Other studies have shown that extracellular enzymes such as collagenase [8], α-clostripain [9] and sialidase [10] are not essential for virulence in the mouse myonecrosis model, although they may play a role in the early stages of disease. Recent experiments have suggested that sialidases may be important in the pathogenesis of animal infections caused by strains of *C. perfringens* type D [11].

In *C. perfringens*, the production of α-toxin, perfringolysin O, collagenase and α-clostripain is controlled either directly or indirectly by the VirSR two-component signal transduction system [12]–[16]. Signal transduction systems enable bacteria to sense changes in their extracellular environment and to respond in a manner that maximizes their chances of survival [17], [18]. Two-component signal transduction pathways generally consist of a membrane-bound sensor histidine kinase, which detects the extracellular or growth phase stimulus, and a cytoplasmic response regulator that often acts as a transcriptional regulator [18]–[21].

The *C. perfringens* type A strain 13 genome contains at least 48 genes that potentially encode proteins involved in signal transduction [22]. As part of a larger study, we focused on *cpe1512*, which appears to be an orphan sensor histidine kinase gene. In this study we report that the *cpe1512* gene product, which we have renamed ReeS (Regulator of extracellular enzymes Sensor), encodes a sensor histidine kinase that is involved in the regulation of extracellular sialidase production, but does not affect virulence in the mouse myonecrosis model.

## Materials and Methods

### Ethics Statement

All mouse virulence trials were conducted in accordance with Victorian State Government regulations and the Monash University Animal Ethics guidelines. These experiments were approved by Monash University SOBS B Animal Ethics Committee. Animals were monitored continually through the experiment to ensure that they did not suffer unduly. Disease symptoms were scored on a scale of 0 (no sign of symptom), 0.5 (moderate disease symptom severity) or 1 (severe affected by disease symptom). When a score of 1 was reached in any symptom other than swelling of the thigh, the mice were euthanized humanely for ethical reasons by CO_2_ asphyxiation, in accordance with our animal ethics approval.

### Bacterial strains, Plasmids, and Media

The *C. perfringens* strain 13 derivative JIR325 [14] was the parent for the construction of the *reeS* mutant. The plasmids used in this study are listed in Table 1. All chemicals and antibiotics were supplied by Sigma unless otherwise stated. Culture media were supplied by Oxoid unless otherwise stated. *C. perfringens* strains were grown in either fluid thioglycolate broth (FTG) (Difco), TPYG (5% (w/v) tryptone, 0.5% (w/v) proteose peptone, 0.3% (w/v) yeast extract, 0.1% (w/v) sodium thioglycolate, 0.37% (w/v) glucose (added after autoclaving)) or on nutrient agar (NA) [23] supplemented with rifampicin (10 µg/ml), nalidixic acid (10 µg/ml), erythromycin (Amresco) (50 µg/ml) or chloramphenicol (30 µg/ml). Agar cultures were grown in an atmosphere of 10% H_2_, 10% CO_2_ and 80% N_2_. *Escherichia coli* cells were incubated under aerobic conditions at 37°C in 2YT broth [24], supplemented with 1.5% (w/v) agar (Oxoid) when required. When applicable, *E. coli* media contained either erythromycin (150 µg/ml) or chloramphenicol (30 µg/ml). Growth was monitored by measuring the optical density (OD) at 600 nm using a WPA Biowave CO8000 cell density meter.

**Table 1:** Bacterial strains and plasmids.

Name	Relevant Characteristics	Origin
**BACTERIAL STRAINS**		
*E. coli*		
DH5α	F^−^Ф80 *lacZ*ΔM15Δ(*lacZYA-argF*)*U169 endA1 recA1 hsdR17* (r_k_^−^ m_k_^−^) *deoR* λ^–^ *thi-1 pho*A *supE44 gyrA96 relA1*	Life Technologies
*C. perfringens*		
JIR325	Rif^R^Nal^R^ derivative of strain 13	[14]
JIR12192	JIR 325Δ*reeS*Ω*erm*(Q)	This Study
JIR12578	JIR12192(pJIR3818)	This Study
**PLASMIDS**		
pJIR418	*E. coli*-*C. perfringens* shuttle vector, carries pIP404 replication origin, Cm^R^ Erm^R^	[31]
pJIR2715	Suicide vector base for *C. perfringens*, Cm^R^ Erm^R^	[30]
pJIR3375	pJIR2715 *(Asp*718/*Bam*HI)ΩJRP3524/JRP3526 (*Asp*718/*Bam*H1, 1.5 kb) PCR product, Cm^R^ Erm^R^	This Study
pJIR3377	pJIR3377 (*Xba*I/*Not*I)ΩJRP3526/JRP3527 (*Xba*I/*Not*I, 1.5 kb) PCR product, Cm^R^ Erm^R^	This Study
pJIR3818	pJIR418 (8 kb *Eco*RI fragment containing *reeS*) Cm^R^ Erm^R^	This Study

### Genetic Manipulations

Plasmid DNA from *E. coli* cells was prepared as described previously [25] or with a Qiagen miniprep purification system. Plasmid DNA for nucleotide sequencing was prepared using a modified PEG precipitation method, as described in the ABI Big Dye Manual (Applied Biosystems). All restriction endonucleases or other enzymes were used according to the manufacturer’s instructions (Roche Diagnostics, New England Biolabs). *C. perfringens* chromosomal DNA was isolated as before [26]. Total RNA was isolated from *C. perfringens* cells as described previously [27], with repeated rounds of DNase I digestion until all of the DNA was removed. Standard methods for the modification, ligation and analysis of plasmid DNA, genomic DNA and PCR products were used [24]. DNA and RNA concentrations were determined using a NanoDrop spectrophotometer (NanoDrop Technologies). DNA sequencing reactions were performed using the PRISM BigDye Terminator Mix (Applied Biosystems). Signal detection was performed on an Applied Biosystems 3730 S Genetic Analyser and sequences analyzed using ContigExpress™ software (Invitrogen). All oligonucleotide primers for PCR or sequencing were obtained from Sigma-Aldrich and are listed in Table 2.

**Table 2:** Oligonucleotide primers.

Name	Sequence (5′-3′)	Function
JRP3524	CGGGGTACCCCATATATATCAAGAAGTATTACTG	PCR 1.5 kb fragment upstream of *reeS*, forward primer
JRP3525	CGGGATCCCTTAAAAATGGAATCATAGAATTAG	PCR 1.5 kb fragment upstream of *reeS*, reverse primer
JRP3526	GCTCTAGACTTATGATTGCACAGTTACC	PCR 1.5 kb fragment downstream of *reeS*, forward primer
JRP3527	ATGCGGCCGCATTAAATTCCTCATCCTATAAC	PCR 1.5 kb fragment downstream of *reeS*, reverse primer
**QRT-PCR Primers**		
JRP2479	CCATCTGTTTTTATATCTGCTCCAGTA	*rpoA*, forward primer
JRP2480	GGAAGGTGAAGGACCAAAAACTATT	*rpoA*, reverse, primer
JRP4209	GCCGATGCTCCTAACAATGATATAG	*nanI*, forward primer
JRP4210	TAGTCCATTATTATTTGTCCTTCATCCC	*nanI*, reverse primer
JRP4416	CATGGAGTGAACCAGAGGATTTAAA	*nanJ*, forward primer
JRP4417	ATTCCCTTTCCTGGTGCAGTT	*nanJ*, reverse primer
JRP4426	GAGGAAAATAAGTTTGCAGAAGTTGTAGT	*nagL*, forward primer
JRP4427	TCATGACTCCAAGGTACTCCATAAAA	*nagL*, reverse primer
JRP5149	ACTGGAGCTATGGTAAGTAATGGA	*nagJ*, forward primer
JRP5150	GGACCAGTCCACATAACTTCTATAC	*nagJ*, reverse primer
JRP5151	AGGATGGGTTGATTCTTTAAGAGA	*nagK*, forward primer
JRP5152	CTTCATAGCTTCCTCATAATTTCCT	*nagK*, reverse primer

*C. perfringens* cells were transformed by electroporation [28] with at least 5 µg of purified plasmid DNA using a BTX ECM-630 Electro Cell Manipulator (BTX Laboratories) with a single electric pulse of 1.8 kV, a resistance of 200 Ω and a capacitance of 25 µF. *E. coli* cells were made chemically competent as before [29] and transformations performed using the heat-shock method [24].

### Construction and Complementation of a *reeS* Mutant

A *reeS* null mutant of strain JIR325 was constructed by allelic exchange, with a double crossover event resulting in the replacement of the *reeS* gene with an *erm*(Q) gene, which confers resistance to erythromycin. The plasmid pJIR3375 was constructed by cloning a 1.5 kb PCR fragment generated by the primers JRP3524 and JRP3525, which bind 1921 bp and 421 bp, respectively, upstream of the *reeS* start codon, from JIR325 genomic DNA (Table 2) into the *Asp*718 and *Bam*HI sites of pJIR2715 [30]. A 1.5 kb PCR fragment generated from JIR325 genomic DNA using the primers JRP3526 and JRP3527, which bind 419 bp and 1912 bp, respectively, downstream of the *reeS* stop codon, then was cloned into the *Xba*I and *Not*I sites of pJIR3375 to give the final suicide vector, pJIR3377 (Table 1). The recombinant plasmids were analyzed by restriction enzyme digestion and sequencing. The *reeS* mutant was constructed by homologous recombination between pJIR3377 and the JIR325 chromosome. Plasmid DNA was introduced by electroporation and erythromycin resistant transformants were selected. The resultant *C. perfringens* strain, JIR12192 (Table 1), was confirmed as a *reeS* mutant by PCR and Southern hybridization (data not shown).

A 6.4 kb *Eco*RI fragment containing the wild-type *reeS* gene, and approximately 2.9 kb of upstream sequence, was cloned into the *C. perfringens*-*E. coli* shuttle plasmid pJIR418, which confers chloramphenicol and erythromycin resistance [31]. The resultant complementation plasmid, pJIR3818, was introduced into the *reeS* mutant JIR12192 to form the complemented *reeS* mutant derivative, JIR12578 (Table 1).

### Microarray Analysis of *C. perfringens* Strains

Microarrays were designed to represent all predicted coding sequences in the *C. perfringens* strain 13 genome, including putative genes present on the cryptic plasmid, pCP13, and some intergenic regions [32]. The cDNA hybridizations and image capture were conducted as described previously [33]. Spot intensities were subjected to statistical analysis using the *Limma* software package for R [34], [35], which firstly normalized per print tip group, then between arrays using Loess normalization. Genes with an expression fold difference of greater than two-times up- or down-regulated with an associated false discovery rate (FDR) of less than 0.05 were considered significant. Microarray data were deposited into the GEO (Gene Expression Omnibus) database with the accession number GSE36786.

### Quantitative Reverse Transcription (QRT)-PCR Analysis of Differentially Expressed Genes

QRT-PCR was used to measure the expression levels from the wild-type, mutant and complemented derivatives and were the average of triplicate reactions performed from cDNA generated from at least three independent biological replicates, as previously described [33]. Statistical analyses were performed using the student’s t-test using GraphPad Prism 5 software. Specific gene expression values were normalized according to the expression of the housekeeping gene, *rpoA*. Signal detection was performed on a Mastercycler ep Realplex real-time PCR machine (Eppendorf) and reactions were confirmed as being the result of a single product by disassociation curve analysis.

### Hyaluronidase and Sialidase Assays

*C. perfringens* cultures were grown in either TPYG or Todd-Hewitt broth (Oxoid) supplemented with 0.1% (w/v) sodium thioglycolate and 0.37% (w/v) glucose after autoclaving. All enzyme assays are expressed as the initial rate of activity per min per mg total protein. Total protein was determined by using the Pierce BCA protein assay kit (Thermo Scientific). Statistical significance was determined using the student’s t-test (GraphPad Prism 5 software) for both hyaluronidase and sialidase activity.

Hyaluronidase activity was determined from six independent biological replicates using the endohexosaminidase-specific fluorogenic substrate, 4-methylumbelliferyl-N-acetyl-β-D-glucosamide (MUG) [36]. Bacterial cell pellets from 15 ml TPYG cultures were washed in sterile DPBS (140 mM NaCl, 2.68 mM KCl, 4.23 mM Na_2_HPO_4_, 10 mM KH_2_PO_4_, pH 7.5) and resuspended in 1 ml of sterile DPBS and treated with 100 µl of lysozyme (10 mg/ml) (Amresco) at 37°C overnight. The lysates were centrifuged for 10 min at 3500 × *g* at room temperature and the supernatant diluted tenfold. Hyaluronidase assays were conducted as described previously [33], with the hydrolysis of MUG measured fluorometrically every two minutes (λ_Ex_ 365 nm, λ_Em_ 412 nm) at room temperature for 60 min in a Tecan Infinite 200 platereader (Tecan). The amount of 4-melthylumbelliferone (4-MU) liberated was determined by comparison to a standard curve of 4-MU (Sigma) ranging from 1 nmol to 100 fmol. Hyaluronidase specific activity is expressed as the amount of 4-MU released per min per mg of total protein.

Sialidase assays were conducted as described previously [10]. Todd-Hewitt broth culture supernatants, derived from three independent biological replicates, were concentrated using Amicon ultra centrifugal devices (Millipore) with a nominal weight cutoff of 30 kDa. Sialidase specific activity is expressed as the increase in absorbance at 620 nm per min per mg of total protein.

### Virulence Trials in Mice

The virulence of *C. perfringens* strains was tested in 6–8 week old female BALB/C mice, as described previously [33], [37]. The virulence of each *C. perfringens* strain was assessed in ten mice using bacterial cells isolated from at least two independent biological replicates. Kaplan-Meier survival curves were generated using GraphPad Prism 5 software and statistical analysis performed using the log rank Mantel-Cox test.

## Results

### Mutation of *reeS* Alters the Expression of Sialidase Genes

Bioinformatic analysis of putative signal transduction systems in *C. perfringens* strain 13 identified the *reeS* gene, which appeared to be a hybrid sensor histidine kinase gene; with no other potential signal transduction proteins encoded either upstream or downstream. The putative ReeS protein had a domain architecture that was similar to sensor histidine kinase proteins. Two potential transmembrane helices were identified within the N-terminal region and potential conserved sensor histidine kinase domains were identified within the C-terminal region of the protein. Putative RE and YYY domains, which have been associated with hybrid sensor kinase proteins [38], [39], were identified within the N-terminal region, although they were located between the two transmembrane domains and therefore may be located in the cell wall region and may not be functional. No potential DNA binding domain, or any other effector domain associated with response regulators, could be identified within the ReeS protein, suggesting that ReeS functions as an orphan sensor histidine kinase, rather than as a hybrid sensor kinase/response regulator.

BLAST searches revealed that ReeS had 26% and 29% amino acid identity to BT3334 and BT4663, respectively; these proteins are predicted to function as hybrid sensor histidine kinase/response regulator fusion proteins in *Bacteroides thetaiotaomicron* [40]. BT3334 and BT4663 are involved in the breakdown of glycosaminoglycans such as hyaluronic acid, chrondroitin sulphate and dermatan sulphate (for BT3334) and heparin sulphate (for BT4663) [40], [41].

To determine if ReeS was involved in the regulation of similar genes in *C. perfringens*, a *reeS* deletion mutant was constructed by homologous recombination and allelic exchange. The genotype of the *reeS* mutant was confirmed by PCR and Southern hybridization (data not shown). Microarray analysis then was used to examine the transcriptome of the *reeS* mutant during exponential growth. Labeled cDNA was generated from RNA isolated from four independent biological replicates of each of the wild-type strain and the *reeS* mutant and hybridized to *C. perfringens* strain 13 specific microarrays [32], including two-dye swaps for each strain. Microarray data were filtered to exclude genes whose expression was less than twofold up- or down-regulated and had an associated FDR value of greater than or equal to 0.05. Using these criteria, we identified 46 genes as being potentially differently regulated in the *reeS* mutant (Table S1), including three putative hyaluronidase-encoding genes, *nagJ*, *nagK* and *nagL* as well as the *nanJ* gene, which encodes the minor sialidase [10]. All of these genes appeared to be down-regulated in the *reeS* mutant (Table S1).

Prior to QRT-PCR validation of the microarray data, the *reeS* mutation was complemented *in trans* with the wild-type *reeS* gene. QRT-PCR was carried out on the hyaluronidase and sialidase encoding genes using RNA isolated from the wild-type, mutant, and complemented strains. The *nanI* gene also was included, since it encodes the major *C. perfringens* sialidase [10]. Due to the variance in spot intensity between arrays, the *nanI* gene (fold ratio of 1.33, FDR* = *0.81) was excluded from analysis in the microarray experiments.

**Figure 1:**
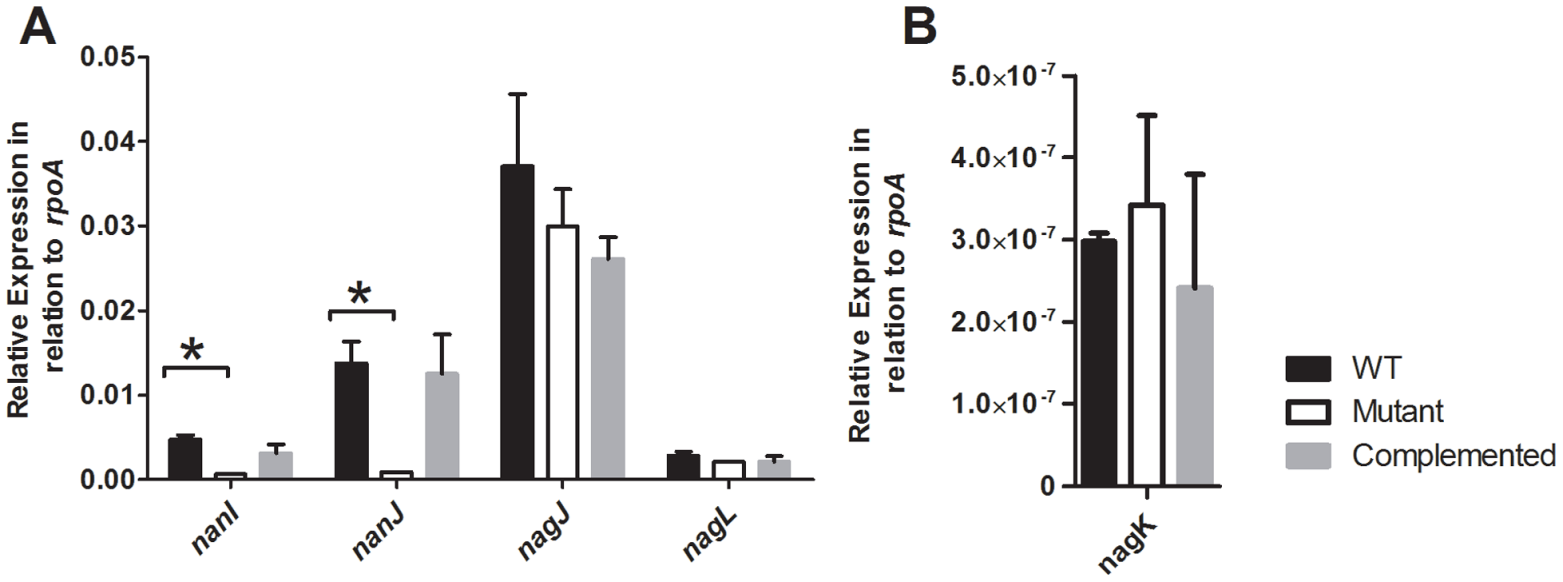
Expression of the sialidase and selected hyaluronidase genes in the wild-type, mutant and complemented strains. RNA was isolated from cells grown in TPYG broth for 4 h, which corresponds to exponential growth phase. The relative expression of the *nanI*, *nanJ*, *nagJ* and *nagL* (A) and *nagK* (B) genes was determined by QRT-PCR. Expression levels are relative to the expression of the housekeeping gene *rpoA* and are the average of three independent biological replicates (± SEM). The asterisk (*) denotes a statistically significant difference (*p*≤0.05) as calculated using the student’s t-test.

Expression of both the *nanI* and *nanJ* genes was shown to be down-regulated in the *reeS* mutant (Figure 1); for *nanJ* this result was consistent with the microarray analysis. Complementation with the wild-type *reeS* gene restored the expression of these genes to levels similar to wild type (Figure 1), confirming that *reeS* was involved in the regulation of the *nanI* and *nanJ* genes. In contrast to the microarray results, which suggested a down-regulation of the hyaluronidase encoding genes (*nagJKL*) in the *reeS* mutant, subsequent QRT-PCR analysis revealed that there was no significant difference in the relative expression of these genes in the *reeS* mutant when compared to the wild type (Figure 1). This discrepancy may be due to signal variation and the multiplicity of testing in the microarrays, which was not accounted for by the FDR measurement.

### Mutation of *reeS* Affects Sialidase Production

**Figure 2:**
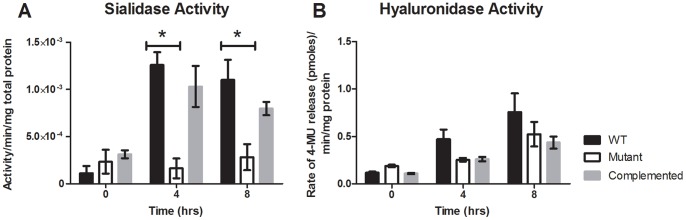
Sialidase and hyaluronidase production by the wild-type, mutant and complemented strains. Quantitative assays were carried out to determine the relative amount of extracellular enzyme production by the wild-type, mutant and complemented strains at time of inoculation and 4 h and 8 h post-inoculation (corresponding to exponential and stationary growth phases, respectively). Sialidase assays were performed using culture supernatants from cultures grown in Todd-Hewitt broth (A). Hyaluronidase activity was determined using the cell lysates from cultures grown in TPYG (B). All results are given as the average of three biological replicates (± SEM) for sialidase assays and six biological replicates for hyaluronidase assays; the asterisk (*) denotes a statistically significant difference (*p*≤0.05) as determined by the student’s t-test.

To determine if the reduced gene expression that was observed in the microarray and QRT-PCR analysis corresponded to an associated decrease in enzyme activity, *in vitro* sialidase and hyaluronidase assays were conducted. Culture supernatants at 0 h (immediately after inoculation), 4 h (exponential growth phase) and 8 h (stationary phase growth) were obtained from three independent biological replicates of each of the wild-type, mutant, and complemented strains and assayed for sialidase and hyaluronidase activity. Consistent with the QRT-PCR analysis, there was a significant decrease in the total sialidase activity of the mutant compared to wild type (Figure 2A); a decrease that was reversed by complementation of the *reeS* mutation (Figure 2A). These results confirmed that ReeS positively regulates sialidase production in *C. perfringens*. In agreement with the QRT-PCR analysis, there was no significant difference in the total hyaluronidase activity of the *reeS* mutant when compared to the wild-type or complemented strains (Figure 2B).

### Mutation of *reeS* does not Affect Virulence

**Figure 3:**
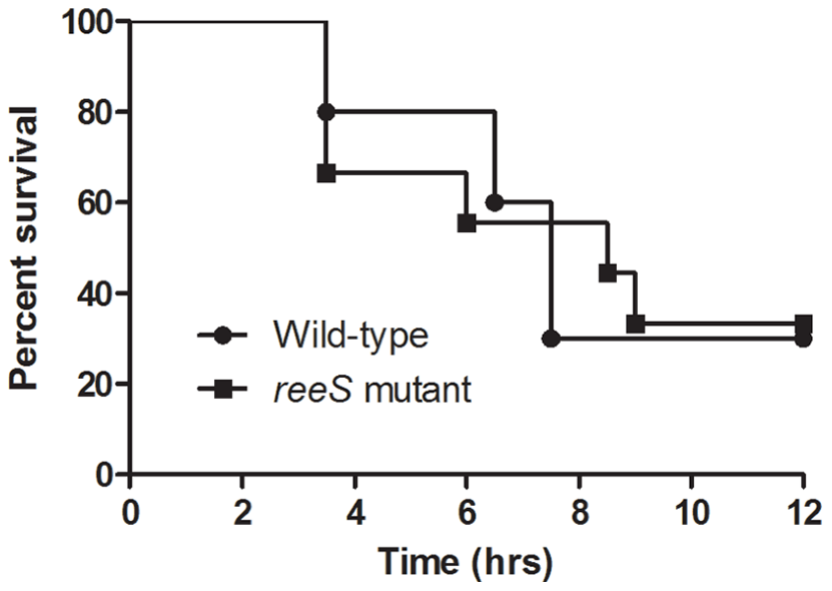
Kaplan-Meyer curves of mice injected with the wild-type or the *reeS* mutant. The wild-type and mutant strains were injected into BALB/c mice (*n* = 10) and their survival was monitored every 30 min for 12 h.

To determine if mutation of the *reeS* gene modulated the ability of the resultant strain to cause disease, ten mice were injected with either the wild-type strain or the *reeS* mutant and the disease outcome recorded. There was no significant difference in the survival of mice infected with these strains (*p = *0.93), as measured by the Mantel-Cox Log-rank test (Figure 3). Furthermore, there was no difference in the severity of the infection caused by the *reeS* mutant when compared to wild-type (data not shown). Since no difference in virulence was observed, the complemented mutant was not tested in the mouse myonecrosis model.

## Discussion

This study reports the identification of a novel orphan sensor histidine kinase, ReeS, that positively regulates sialidase expression in *C. perfringens*. QRT-PCR experiments confirmed that transcription of the sialidase-encoding *nanI* and *nanJ* genes was reduced in a *reeS* mutant; complementation restored gene expression to approximately wild-type levels. These transcriptomic data were confirmed by corresponding changes in the level of extracellular sialidase activity, providing clear evidence that ReeS controls the production of extracellular sialidases in *C. perfringens* strain 13.

**Figure 4:**
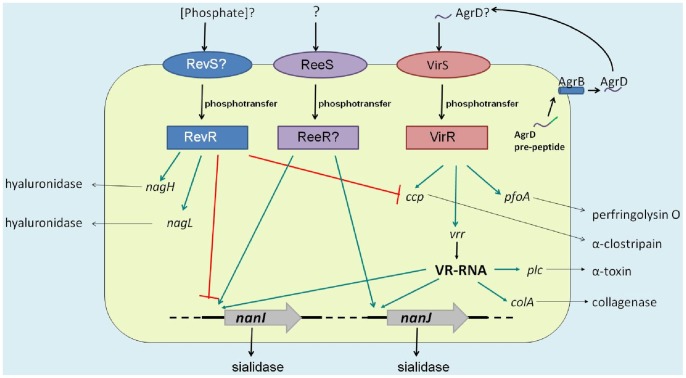
The proposed model of sialidase gene regulation by ReeS, RevR and VirSR. The different effects of the VirSR, RevR and ReeS systems on extracellular enzyme/toxin production are shown. The ReeS sensor kinase positively regulates *nanI* and *nanJ* sialidase gene expression by an unknown response regulator, ReeR. By contrast, upon detection of an unknown signal (possibly phosphate) an unidentified sensor histidine kinase protein, RevS, acts as a phsophodonor of RevR, which positively regulates *nanJ* expression but negatively regulates *nanI* expression. Finally, it is proposed that in response to the detection of the processed AgrD peptide phosphotransfer occurs between VirS and VirR. Phosphorylated VirR acts as a positive regulator of the *vrr* gene, encoding VR-RNA, which subsequently acts as a positive regulator of *nanI* and *nanJ*. Green lines indicate positive regulation, whereas red lines indicate negative regulation.

The regulation of sialidase production in *C. perfringens* is a complex process, which with the identification of ReeS now appears to involve three independent global regulatory systems (Figure 4). The best studied is the VirSR two-component signal transduction system. This network regulates the expression of the *nanI* and *nanJ* genes via the VirR-regulated RNA molecule, VR-RNA [32], which also regulates the expression of other virulence genes such as *plc* (encoding α-toxin) and *colA* (encoding collagenase) [12]. Further control of sialidase gene expression occurs via the RevR response regulator [33]. In a *revR* mutant, expression of the *nanI* gene is up-regulated, whereas the *nanJ* gene is down-regulated (Figure 4). In addition to regulating sialidase production, RevR negatively regulates the expression of the *ccp* gene (encoding α-clostripain) and positively regulates the expression of the *nagH* and *nagL* genes (Figure 4) [33]. Furthermore, the regulation of these genes by RevR occurs independently of the VirSR systems [33].

In the *reeS* mutant, the expression of the known VirSR regulated genes, *pfoA* and *plc*, was unaffected by the *reeS* mutation (data not shown), suggesting that the regulation of sialidase production by ReeS also was independent of the VirSR network. Furthermore, the expression of the known RevR-regulated genes, *nagH* and *ccp*, was unaffected by the *reeS* mutation and the regulation of *nanI* and *nanJ* is different. ReeS was shown to positively regulate both *nanI* and *nanJ*, whereas RevR positively regulates *nanJ* expression, but negatively regulate *nanI* expression [33]. These results provide evidence that ReeS controls sialidase production independently of both the VirSR system and RevR.

The complexity of the regulation of sialidase production is further emphasized by the different signals detected by VirSR and RevR. Recent evidence suggests that an *agr*–like quorum sensing pathway may control the production of α-toxin, perfringolysin O and β2-toxin in *C. perfringens* [42]–[45] and has led to the proposal that an autoinducing peptide encoded by *agrD* activates VirS autophosphorylation [42]. These data suggest that that the control of sialidase production by the VirSR system is part of a larger quorum sensing pathway. Although the stimulus for the cognate sensor histidine kinase of RevR has not been determined, it has been suggested that RevR operates as a potential PhoB homologue based on the high sequence similarity between RevR and PhoB from *Clostridium kluyveri* (62% amino acid identity) and the close proximity of *revR* to a putative phosphate operon [33]. If RevR functions as a PhoB homologue, the regulation of sialidase genes by RevR would be expected to occur in response to extracellular phosphate levels. At present the external signal that activates ReeS is unknown, but based on the 26% amino acid identity between ReeS and the hybrid sensor histidine kinase/response regulators, BT3334 and BT4663, which are thought to be involved with the sensing of extracellular glycoaminoglycans [41], it is possible that ReeS may function in a similar manner. Alternatively, it may be activated by sialic acid-containing glycoproteins. The variety of different stimuli that may activate VirSR or RevR systems suggests that sialidase production is important for the survival of the organism, possibly for the acquisition of nutrients.

In other bacteria sialidases are important virulence factors [46], [47]. However, using the mouse myonecrosis model we did not observe any difference in the virulence of the *reeS* mutant compared with the wild-type strain, in agreement with a previous study which showed that sialidase production is not essential for virulence [10]. These results may reflect the nature of the myonecrosis model, which will not reveal virulence factors required for the very early stages of a natural infection. Previous studies showed that sialidases enhance the myotoxic action of α-toxin in mice [48] and more recent studies have shown that sialidase production, mainly NanI, is important for the binding and cytotoxic effects of ε-toxin, the primary virulence factor of *C. perfringens* type D infections [11]. This evidence suggests a role of sialidases in the development of *C. perfringens* infections. Further studies are required to determine if ReeS is required for the early stages of a myonecrotic *C. perfringens* infection.

We attempted to identify the effector molecule by which ReeS activates transcription, but bioinformatic searches of the region surrounding the *reeS* gene did not reveal any genes encoding proteins putatively involved in signal transduction. No DNA binding domain, or any other effector domain associated with response regulators, could be identified within the ReeS amino acid sequence. These results suggest that ReeS is most likely to function as an orphan sensor histidine kinase, with the identity of its cognate response regulator yet to be determined. Neither BT3334 or BT4663 have a cognate response regulator protein, therefore bioinformatic searches did not yield any potential response regulator partners for ReeS. Further studies are required to identify the cognate response regulator partner, or partners, of ReeS.

Finally, the discrepancy between the microarray data and QRT-PCR results for the putative hyaluronidase encoding genes *nagJKL* appeared to be due to changes in the individual spot intensities in two out of the four arrays. This finding emphasizes the importance of the validation of key microarray results by QRT-PCR analysis or transcriptome sequencing and the tendency for false positives, due to the multiplicity of testing of large data sets in microarray experiments. A similar observation was reported in our RevR studies, in which microarray analysis suggested that the *colA* gene (encoding collagenase) was up-regulated in the *revR* mutant, but subsequent QRT-PCR assays and *in vitro* collagenase assays revealed no difference between the mutant and wild type [33].

## Conclusions

*C. perfringens* is a versatile organism, able to survive within different ecological niches from the soil, sewage and the human and animal gastrointestinal tracts [49]. The *C. perfringens* strain 13 genome has been shown to encode a total of 48 different genes whose products are predicted to be involved in signal transduction [22], yet the majority of the research conducted on signal transduction in *C. perfringens* has focused on the VirSR two-component signal transduction system. Here we have identified the orphan sensor histidine kinase ReeS and shown that it is involved in the transcriptional activation of genes encoding the major and minor sialidase proteins, NanI and NanJ. Previous work has suggested that the global VirSR two-component signal transduction system and the RevR response regulator are involved in the transcriptional regulation of both the *nanI* and *nanJ* genes [33], [42]. This paper shows that sialidase production is also regulated by ReeS in a manner that is independent to both VirSR and RevR, adding further complexity to the global gene regulation network of *C. perfringens*.

## Supporting Information

Table S1 **Differently regulated genes in the** ***reeS*** **mutant.**
(PDF)
