# Supplementary material for: Regulation of Sialidase Production in Clostridium perfringens by the Orphan Sensor Histidine Kinase ReeS
Source: PLoS One. 2013 Sep 4;8(9):e73525. doi: 10.1371/journal.pone.0073525 (PMC3762733; doi:10.1371/journal.pone.0073525)
Supplement: Table S1 — Differently regulated genes in the reeS mutant. (PDF) [file pone.0073525.s001.pdf]

Table S1: Differently regulated genes in the *reeS* mutant. Microarrays were designed to include all potential protein-coding sequences from the strain 13 genome, including the native plasmid, pCP13 [1,2] and were hybridized with labeled cDNA generated from the wild-type and *reeS* mutant. Fold ratios are expressed as a ratio of *reeS* mutant to wild-type expression.

Locus Tag	Function	Fold ratio	FDR
CPE2182	conserved hypothetical protein	0.03	4.0E-03
PCP06	probable transposase	0.04	3.7E-04
CPE1234	putative hyaluronidase (NagJ)	0.05	3.3E-04
CPE1761	probable transcription initiation factor SigG	0.05	3.3E-04
CPE1343	probable galactoside ABC transporter (permease protein)	0.05	1.8E-03
PCP08	hypothetical protein	0.05	3.3E-04
CPE0554	hypothetical protein	0.05	3.5E-03
PCP07	probable ABC transporter	0.05	9.7E-04
CPE1334	serine protein kinase	0.05	1.1E-02
CPE1808	probable integrase/recombinase	0.05	3.3E-04
CPE1753	stage IV sporulation protein A	0.06	7.7E-03
CPE1341	probable galactoside ABC transporter (D-galactose/D-glucose-binding protein)	0.06	1.1E-02
CPE1874	probable general stress protein	0.06	9.7E-04
CPE0113	hypothetical protein	0.06	4.3E-02
CPE1257	conserved hypothetical protein	0.08	4.0E-03
CPE1667	probable single-strand binding protein	0.08	2.0E-03
CPE0206	hypothetical protein	0.08	2.0E-02
PCP53	hypothetical protein	0.08	1.0E-03
CPE0818	endo-beta-N-acetylglucosaminidase	0.08	9.7E-04
CPE2632	probable PTS system,, mannose-specific, component IIAB	0.08	8.1E-04
CPE0068	glucose-1-phosphate adenylyltransferase	0.09	1.2E-03
CPE2218	probable spore coat protein	0.09	9.6E-03
CPE0886	hypothetical protein	0.09	4.0E-03
PCP57	collagen binding protein (Cna)	0.09	1.0E-02
CPE1142	hypothetical protein	0.09	1.9E-03
CPE2288	prepilin peptidase dependent protein A	0.10	2.4E-02
IGR31	intergenomic region	0.10	9.2E-03
CPE2295	signal peptidase type I	0.10	4.2E-03
CPE0114	hypothetical protein	0.10	1.9E-03

CPE2226	hypothetical protein	0.10	6.2E-03
CPE1523	putative hyaluronidase (NagL)	0.10	3.1E-03
CPE2179	conserved hypothetical protein	0.11	1.2E-03
CPE0069	glycogen biosynthesis protein (GlgD)	0.11	3.1E-02
CPE2263	hypothetical protein	0.11	4.5E-03
CPE2215	hypothetical protein	0.11	7.7E-03
CPE2631	probable PTS system, mannose-specific, component IIC	0.11	9.8E-03
CPE1949	hypothetical protein	0.12	2.6E-02
CPE2040	stage II sporulation protein P	0.12	3.2E-02
CPE0105	hypothetical protein	0.12	2.3E-02
CPE2630	probable mannose permease IIm	0.12	6.6E-03
CPE2504	hypothetical protein	0.13	4.3E-02
PCP50	hypothetical protein	0.13	2.6E-02
CPE2261	hypothetical protein	0.13	2.2E-02
PCP35	hypothetical protein	0.13	9.2E-03
CPE1266	beta-galactosidase	0.14	4.5E-03
PCP09	hypothetical protein	0.14	2.1E-02
PCP51	hypothetical protein	0.14	9.7E-04
CPE1502	hypothetical protein 10C	0.14	1.9E-03
PCP44	hypothetical protein	0.14	1.5E-02
CPE2353	probable spore cortex-lytic enzyme	0.15	3.2E-02
CPE0430	probable type I signal peptidase	0.15	4.2E-03
CPE1875	conserved hypothetical protein	0.15	2.0E-02
CPE0507	hypothetical protein	0.15	2.3E-02
CPE1920	hypothetical protein	0.15	2.8E-02
CPE2146	hypothetical protein	0.16	1.1E-02
PCP54	hypothetical protein	0.16	2.0E-03
CPE0578	probable ABC transporter (permease)	0.16	1.2E-02
PCP43	hypothetical protein	0.17	4.0E-02
CPE2135	hypothetical protein	0.18	1.8E-02
CPE2050	anti-sigma F factor antagonist (stage II sporulation protein AA)	0.18	1.5E-02
CPE0576	probable ABC transporter (binding protein)	0.18	1.9E-03
CPE1368	conserved hypothetical protein	0.19	2.6E-02
PCP36	hypothetical protein	0.19	6.6E-03
CPE1342	probable galactoside ABC transporter (ATP-binding protein)	0.19	9.2E-03
PCP34	hypothetical protein	0.20	2.5E-02
CPE2049	anti-sigma F factor antagonist (stage II sporulation protein AB)	0.20	9.2E-03
CPE2473	stage II sporulation protein E	0.20	9.2E-03
CPE1279	putative hyaluronidase (NagK)	0.21	5.5E-03

CPE1251	hypothetical protein	0.21	9.2E-03
CPE1353	hypothetical protein	0.22	2.6E-02
CPE1876	conserved hypothetical protein	0.22	2.2E-02
CPE0422	conserved hypothetical protein	0.22	2.8E-02
PCP30	hypothetical protein	0.23	1.8E-02
CPE0553	exo-alpha-sialidase (NanJ)	0.23	8.1E-02
CPE2445	conserved hypothetical protein	0.24	3.2E-02
CPE1256	NADPH-dependent butanol dehydrogenase	0.24	4.9E-02
CPE2649	conserved hypothetical protein	0.25	2.6E-02
PCP33	conserved hypothetical protein	0.26	1.5E-02
PCP60	conserved hypothetical protein	0.27	2.1E-02
PCP55	potential Spo0A homolog	0.28	2.5E-02
IGR111	intergenomic region	0.28	3.6E-02
CPE0421	probable glycerophosphoryl diester phosphodiesterase	0.28	1.7E-02
CPE0291	holo-(acyl-carrier-protein) synthase	0.29	9.2E-03
CPE0577	probable ABC transporter (permease)	0.30	4.1E-02
PCP52	hypothetical protein	0.30	2.1E-02
PCP32	probable resolvase	0.30	2.7E-02
CPE1663	conserved hypothetical protein	0.30	3.8E-02
CPE0471	hypothetical protein	0.32	2.6E-02
PCP58	PemK-like protein	0.32	1.1E-02
CPE2574	hypothetical protein	0.33	2.1E-02
CPE1362	conserved hypothetical protein	0.36	2.8E-02
CPE1030	conserved hypothetical protein	0.37	2.2E-02
PCP31	hypothetical protein	0.38	2.1E-02
PCP12	hypothetical protein	0.38	4.9E-02
CPE2342	probable maltose ABC transporter (permease protein)	0.39	2.1E-02
CPE1596	probable acetylxylan esterase	0.42	4.9E-02
CPE2058	glutamate decarboxylase	2.91	2.4E-02
IGR22	intergenomic region	10.36	4.8E-02

References

1. Shimizu T, Ohtani K, Hirakawa H, Ohshima K, Yamashita A, et al. (2002) Complete genome sequence of *Clostridium perfringens*, an anaerobic flesh-eater. Proc Natl Acad Sci U S A 99: 996-1001.
2. Ohtani K, Hirakawa H, Tashiro K, Yoshizawa S, Kuhara S, et al. (2010) Identification of a two-component VirR/VirS regulon in *Clostridium perfringens*. Anaerobe 16: 258-264.
